# Associations between chronotype, *MTNR1B* genotype and risk of type 2 diabetes in UK Biobank

**DOI:** 10.1111/joim.12994

**Published:** 2019-11-06

**Authors:** X. Tan, D.‐M. Ciuculete, H.B. Schiöth, C. Benedict

**Affiliations:** ^1^ Department of Neuroscience Uppsala University Uppsala Sweden; ^2^ Institute for Translational Medicine and Biotechnology Sechenov First Moscow State Medical University Moscow Russia

**Keywords:** chronotype, genetic risk, melatonin receptor 1B polymorphism, type 2 diabetes, UK Biobank

## Abstract

**Objective:**

To examine the association between the *MTNR1B* G risk allele, type 2 diabetes (T2D) and chronotype in the UK Biobank.

**Methods:**

Data from the baseline investigation of the UK Biobank were utilized (*n* = 337 083 White British; mean age: 56.9 years; 54% women). *MTNR1B* rs10830963 was directly genotyped [CC (reference group), CG and GG]. Chronotype was divided into four categories: definitely morning (reference group); more morning than evening; more evening than morning; and definitely evening. Logistic regression analyses were performed to estimate odds ratios and 95% confidence intervals (CIs) for T2D, controlling for age, sex and other confounders.

**Results:**

Carriers of the rs10830963 risk allele had a higher risk of T2D [CG vs. CC: OR (95% CI) 1.10 (1.07, 1.15); GG vs. CC: 1.21 (1.14, 1.29)]. Compared with definitely morning chronotype, participants with definitely evening chronotype exhibited the highest risk of T2D [1.25 (1.17, 1.33)]. Despite a nonsignificant interaction between chronotype and the risk allele [0.98 (0.94, 1.01), *P* = 0.176 for interaction term], we found that definitely evening chronotype (vs. definitely morning) was linked with a higher risk of T2D amongst CC and CG but not GG carriers. Additionally, we saw that the GG genotype (vs. CC) was associated with a higher risk of T2D across all chronotype categories, except for definitely evening.

**Conclusion:**

Our findings suggest that the *MTNR1B* G risk allele and late chronotype increase the risk of T2D. The association between late chronotype and higher risk of T2D appears to vary across *MTNR1B* rs10830963 genotypes.

## Graphical abstract

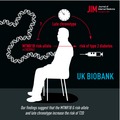

## Introduction

The pineal gland hormone melatonin normally rises in blood 2–3 h before bedtime, remains elevated during the first hours of the night and returns to baseline before the end of the nocturnal sleep period 1. Whilst melatonin is well known for its sleep‐promoting effect 2, it also plays an important role in the regulation of blood glucose levels. For instance, the activation of melatonin receptor 1B (MTNR1B) inhibits glucose‐stimulated insulin secretion by pancreatic beta cells. This can lead to hyperglycaemia 3, 4. As suggested previously, the inhibitory effect of melatonin on the pancreatic insulin release appears to be more pronounced in carriers of a common variant in the *MTNR1B* gene (rs10830963) 5. Besides, carriers of this single nucleotide polymorphism (SNP) exhibit a 1.37‐h delayed dim‐light melatonin offset in the morning 6. A spillover of melatonin into the next day may increase the risk of hyperglycaemia, especially when consuming carbohydrates during early morning hours. Altogether, this could explain the robust association between the *MTNR1B* and the risk to develop type 2 diabetes (T2D) 7, 8.

Noteworthy, a person’s chronotype, which is strongly connected to the endogenous circadian rhythm of the pineal gland hormone melatonin, that is the hormonal ligand of *MTNR1B*, may alter the strength of the association between the *MTNR1B* risk allele and T2D. Contrary to what has been seen in the general population (i.e. late chronotype is linked with an increased risk of diabetes 9, a previous study found that early risers carrying the rs10830963 risk G allele exhibited higher odds of having T2D, compared with carriers with late chronotype 6. However, these findings were based on a relatively small sample (*n* = 1513) and did not take into account confounders, such as sleep duration and insomnia symptoms. Given that both extreme chronotype and the *MTNR1B* risk allele increase the risk of diabetes 7, 9, it is important to study their possible interaction in large cohorts, whilst adjusting for potential confounders. With this in mind, we examined in one of the largest cohort studies worldwide ‐the UK Biobank‐ the association between the *MTNR1B* rs10830963 G risk allele, chronotype and T2D.

## Research design and methods

### Study population

Data from the UK Biobank baseline investigation (2006–2010) were utilized. The UK Biobank enrolled over 500 000 individuals aged between 40 and 69 years from the United Kingdom. Initially, 408 903 White British individuals with available genetic data were considered for the present study. Specifically, participants who self‐reported as not being of British descent (UK Biobank field ID 21000) and those who were not classified as *White British* by principal components of ancestry analysis (ID 22006) were excluded. To minimize the effect of relatedness, cases used in principal components calculation (ID 22020) were applied, yielding a sample of 337 488 participants. Further exclusions were made based on sample failure (ID 22010) and quality‐control failure of the samples genotyped with UK BiLEVE (ID 22050 and ID 22051). Following these exclusions, 337,083 White British individuals with variables of interest were available for the present analysis. The UK Biobank received ethics approval from the National Health Service Research Ethics Service (reference 11/NW/0382), and participants gave informed consent.

### Genotyping

Participants were genotyped on the Affymetrix UK Biobank Lung Exome Evaluation (UK BiLEVE) Axiom array or the Applied Biosystems UK Biobank Axiom Array. Quality control and imputation using the Haplotype Reference Consortium, UK10K and 1000 Genomes phase 3 reference panels were conducted centrally at the UK Biobank, resulting in a total of 96 million SNPs 10. *MTNR1B* rs10830963 SNP (chromosome 16) was amongst the directly genotyped SNPs of the UK Biobank. Testing for Hardy–Weinberg equilibrium (using a chi‐squared test, 1 df) revealed that the SNP did not deviate from expected genotype proportion (*P*‐value > 10^−20^, Fisher’s exact test) 11. The minor allele frequency of the rs10830963 G risk allele was 27.5%. As described elsewhere 6, an additive genetic model was assumed for the SNP on the risk of T2D.

### Chronotype

Chronotype (ID 1180) was asked as ‘Do you consider yourself to be?’ with response options of ‘Definitely a “morning” person’, ‘More a “morning” than an “evening” person’, ‘More an “evening” than a “morning” person’, ‘Definitely an “evening” person’, ‘Do not know’ and ‘Prefer not to answer’. ‘Do not know’ or ‘Prefer not to answer’ responses were treated as missing values. The question is very similar to the last question in the Morningness–Eveningness Questionnaire, which asks ‘One hears about “morning” and “evening” types of people. Which one of these types do you consider yourself to be?’ and had the response options ‘Definitely a “morning” type, Rather more a “morning” than an “evening” type, Rather more an “evening” type than a “morning” type, Definitely an “evening” type’ 12.

### Prevalence of T2D

As described elsewhere 13, probable T2D was defined using a validated algorithm based on self‐reported disease, medication and T2D diagnosis in medical history.

### Confounders

Age (ID 21022), sex (ID 31), body mass index (BMI) (ID 21001), systolic blood pressure (ID 4080, if missing ID 93), smoking status (ID 20116, current, former and never), alcohol intake frequency (ID 1558, six categories from never to daily or almost daily), region of test centre (ID 93, re‐categorized into England, Scotland and Wales), Townsend index reflecting socio‐economic status (ID 189), genetic principal components of ancestry (ID 22009, first ten columns), insomnia (ID 1200, never/rarely, sometimes and usually) and self‐reported sleep duration per day (ID 1160, input as a whole number 1 h≤ and ≤23 h) were included as confounders in our analyses.

### Statistical analysis

Missing values were found in BMI (0.3% of total cases), systolic blood pressure (0.1%), smoking status (0.4%), alcohol intake frequency (0.1%), Townsend index (0.1%), insomnia (0.1%), self‐reported sleep duration (0.5%) and chronotype (10.7%). Multivariate imputation using linear regression method with Markov Chain Monte Carlo procedure was thus performed 14. All other variables involved in the final analyses were used as predictors in the imputation of each variable. Imputation was based on the assumption that data were missing at random. We performed five repetitions of imputations in a model involving all variables in this study. The imputed values were compared with the observed values to evaluate the performance of the imputation. Variables without missing values (age, sex, test centre, prevalence of T2D, rs10830963 genotype and principal components of ancestry) were involved in the imputation as predictors only.

To investigate independent associations of the rs10830963 genotype and chronotype with the risk of T2D, logistic regression analyses were performed. The primary analysis was adjusted for sex and age (Model 1). Model 2 was additionally adjusted for chronotype (or rs10830963 genotype when analysing the association between chronotype and T2D). The third model (Model 3) was adjusted for sex, age, sleep duration and insomnia. The last model (Model 4) was adjusted for age, sex, chronotype (or rs10830963 genotype when analysing the association between chronotype and T2D), sleep duration, insomnia, BMI, systolic blood pressure, smoking, alcohol intake frequency, test centre, principal components of ancestry and the Townsend index. In order to compare the goodness of fit between statistical models, we computed the Nagelkerke’s *R*‐squared value for each logistic regression model. A higher *R*‐squared value reflects better fitness of the logistic regression model 15. The association between the interaction term ‘rs10830963 genotype * chronotype’ and T2D was examined whilst controlling for age and sex, as well as in the fully adjusted model (Model 4). Baseline comparisons between participants with and without T2D were performed by one‐way analysis of variance or chi‐squared test. Two‐tailed tests were used throughout, and a probability level of 0.05 was considered significant. All statistical analyses were performed using SPSS, version 24.0 (SPSS Inc, Chicago, IL, USA).

## Results

General characteristics of the population, separated by T2D status, are shown in Table 1. The estimated prevalence of T2D was 4.3%. Compared with nondiabetic participants, subjects with T2D were, for instance, characterized by a higher BMI and elevated systolic blood pressure and were more likely to be a current or former smoker. The distribution of chronotype across the rs10830963 genotype is presented in Table 2.

**Table 1:** Baseline characteristics of the UK Biobank population, separated by type 2 diabetes status

Characteristics	Total (*n* = 337 083)	Non‐T2D (*n* = 322 558)	T2D (*n* = 14 525)	*P* *
Age, y	56.9 ± 8.0	56.7 ± 8.0	60.7 ± 6.5	0.0E0
Sex, *n* (%)				0.0E0
Women	181 026 (53.7)	175 846 (54.5)	5180 (35.7)	
Men	156 057 (46.3)	146 712 (45.5)	9345 (64.3)	
BMI, kg/m^2^ a	27.4 ± 4.7	27.2 ± 4.6	31.8 ± 5.8	0.0E0
Systolic blood pressure, mmHg^a^	140.2 ± 19.7	140.0 ± 19.7	144.3 ± 18.4	8.6811E‐144
Smoking status, *n* (%)				2.2045E‐249
Current	33 921 (10.1)	32 356 (10.0)	1565 (10.8)	
Former	118 365 (35.1)	111 493 (34.6)	6872 (47.3)	
Never	183 623 (54.5)	177 638 (55.1)	5985 (41.2)	
Missing data	1174 (0.3)	1071 (0.3)	103 (0.7)	
Alcohol intake frequency, *n* (%)				0.0E0
Daily or almost daily	72 181 (21.4)	69 870 (21.7)	2311 (15.9)	
3–4 times per week	81 360 (24.1)	78 968 (24.5)	2392 (16.5)	
1–2 times per week	88 660 (26.3)	85 095 (26.4)	3565 (24.5)	
1–3 times per month	37 316 (11.1)	35 475 (11.0)	1841 (12.7)	
Special occasions only	35 366 (10.5)	32 804 (10.2)	2562 (17.6)	
Never	21 964 (6.5)	20 127 (6.2)	1837 (12.6)	
Missing data	236 (0.1)	219 (0.1)	17 (0.1)	
Region of test centre, *n* (%)				0.0042
England	297 287 (88.2)	284 480 (88.2)	12 807 (88.2)	
Wales	14 807 (4.4)	14 103 (4.4)	704 (4.8)	
Scotland	24 989 (7.4)	23 975 (7.4)	1014 (7.0)	
Townsend index^b^	−1.6 ± 2.9	−1.6 ± 2.9	−0.8 ± 3.3	3.1073E‐238
Insomnia, *n* (%)				4.2514E‐120
Never/rarely	80 404 (23.9)	77 468 (24.0)	2936 (20.2)	
Sometimes	160 889 (47.7)	154 674 (48.0)	6215 (42.8)	
Usually	95 556 (28.3)	90 203 (28.0)	5353 (36.9)	
Missing data	234 (0.1)	213 (0.1)	21 (0.1)	
Self‐reported sleep duration, h/d^c^	7.2 ± 1.1	7.2 ± 1.1	7.3 ± 1.3	9.209E‐21
Chronotype, *n* (%)				7.9469E‐38
Definitely morning	79 955 (23.7)	76 259 (23.6)	3696 (25.4)	
More morning than evening	109 408 (32.5)	105 246 (32.6)	4162 (28.7)	
More evening than morning	85 660 (25.4)	82 016 (25.4)	3644 (25.1)	
Definitely evening	26 016 (7.7)	24 588 (7.6)	1428 (9.8)	
Missing data	36 044 (10.7)	34 449 (10.7)	1595 (11.0)	
*MTNR1B* rs10830963 genotype, *n* (%)				6.0966E‐11
CC	177 213 (52.6)	169 954 (52.7)	7259 (50.0)	
CG	134 193 (39.8)	128 167 (39.7)	6026 (41.5)	
GG	25 677 (7.6)	24 437 (7.6)	1240 (8.5)	

Data are presented as mean ± SD unless otherwise specified.

non‐T2D *n* = 321 574; T2D *n* = 14 416.

a non‐T2D *n* = 322 277; T2D *n* = 14 498.

b non‐T2D *n* = 322 176; T2D *n* = 14 509.

c non‐T2D *n* = 320 926, T2D *n* = 14 367.

* Difference between non‐T2D and T2D participants, one‐way ANOVA or chi‐squared test.

**Table 2:** Crosstab between chronotype and *MTNR1B* rs10830963 genotype

Chronotype	rs10830963 genotype		
	CC	CG	GG
	*n* (%)	*n* (%)	*n* (%)
Definitely morning	41 691 (23.5)	32 155 (24.0)	6109 (23.8)
More morning than evening	57 593 (32.5)	43 491 (32.4)	8324 (32.4)
More evening than morning	45 249 (25.5)	33 825 (25.2)	6586 (25.6)
Definitely evening	13 701 (7.7)	10 429 (7.8)	1886 (7.3)
Missing data	18 979 (10.7)	14 293 (10.7)	2772 (10.8)

The main effects of the *MTNR1B* gene and chronotype on the risk of T2D are presented in Table 3. Compared with noncarriers, carriers of rs10830963 G risk allele had a higher risk of T2D [OR (95% CI), CG vs. CC (reference group): 1.10 (1.07, 1.14), *P* = 4.01E‐8; GG vs. CC: 1.19 (1.12, 1.27), *P* = 3.33E‐8, Model 1]. The association between rs10830963 and risk of T2D persisted when adjusting for chronotype, insomnia and other confounders [CG vs. CC: 1.10 (1.07, 1.15), *P* = 1.13E‐7; GG vs. CC: 1.21 (1.14, 1.29), *P* = 5.60E‐9, Model 4]. A separate logistic regression revealed that compared with those with a definitely morning chronotype, definitely evening chronotype was associated with the highest risk of T2D [1.38 (1.29, 1.46), *P* = 0.0E0, Model 1]. The link between definitely evening chronotype and the highest risk of T2D persisted even after controlling for multiple confounders [definitely evening vs. definitely morning: 1.25 (1.17, 1.33), *P* = 7.08E‐11, Model 4]. We also found that those who reported having ‘more morning than evening’ chronotype had a lower risk of T2D [0.84 (0.80, 0.89), *P* = 2.31E‐10, Model 1]; however, this effect did not reach statistical significance in the fully adjusted model (i.e. Model 4). Note that above‐mentioned results were similar on sensitivity analysis using un‐imputed data only (i.e. data from participants without missing values; *n* = 297 091; data shown in the supplementary Table S1).

**Table 3:** Odds ratios and 95% CIs for type 2 diabetes, separated by rs10830963 genotype and chronotype

Exposure	Model 1		*R* ^2^	Model 2		*R* ^2^	Model 3		*R* ^2^	Model 4		*R* ^2^
	OR (95% CI)	*P* *		OR (95% CI)	*P* *		OR (95% CI)	*P* *		OR (95% CI)	*P* *	
rs10830963 genotype												
CC	1		0.056	1		0.059	1		0.063	1		0.184
CG	1.10 (1.07, 1.14)	4.0051E‐8		1.10 (1.07, 1.14)	3.8816E‐8		1.10 (1.08, 1.12)	4.1039E‐8		1.10 (1.07, 1.15)	1.1282E‐7	
GG	1.19 (1.12, 1.27)	3.3262E‐8		1.19 (1.12, 1.27)	2.5537E‐8		1.19 (1.16, 1.23)	2.7255E‐8		1.21 (1.14, 1.29)	5.6032E‐9	
Chronotype												
Definitely morning	1		0.058	1		0.059	1		.065	1		0.184
More morning than evening	0.84 (0.80, 0.89)	2.3055E‐10		0.84 (0.80, 0.89)	2.8277E‐10		0.84 (0.80, 0.88)	9.9992E‐11		0.96 (0.91, 1.01)	0.088	
More evening than morning	1.01 (0.96, 1.05)	0.791		1.01 (0.96, 1.05)	0.769		1.00 (0.95, 1.04)	0.833		1.06 (1.01, 1.12)	0.012	
Definitely evening	1.38 (1.29, 1.46)	0.0E0		1.37 (1.29, 1.46)	0.0E0		1.34 (1.26, 1.43)	0.0E0		1.25 (1.17, 1.33)	7.0805E‐11	

Model 1: adjusted for age and sex.

Model 2: adjusted for confounders in Model 1 + self‐reported chronotype (or rs10830963 genotype when investigating the association between self‐reported chronotype and T2D).

Model 3: adjusted for confounders in Model 1 + self‐reported sleep duration + insomnia.

Model 4: adjusted for confounders in Model 2 + self‐reported sleep duration + insomnia + BMI + systolic blood pressure + smoking + alcohol intake frequency + test centre + principal components of ancestry + Townsend index.

* Compared to the reference group (derived from logistic regression analysis). In order to compare the goodness of fit between statistical models, we computed the Nagelkerke’s *R*‐squared value for each logistic regression model. A higher *R*‐squared value reflects better fitness of the logistic regression model 15.

No significant genotype–chronotype interaction regarding the risk of T2D was detected [0.98 (0.94, 1.01), *P* = 0.176, Model 4; *P* = 0.339 when adjusting for age, sex, chronotype and rs10830963 genotype]. When stratifying our analysis by chronotype (Table 4a), we found that carriers with one copy of the G risk allele (i.e. CG) reporting either a ‘definitely morning’ or ‘more morning than evening’ chronotype had a higher risk of T2D compared with noncarriers (i.e. CC). In contrast, no differences in the T2D risk were found between CG and CC carriers in the later chronotype categories (i.e. ‘more evening than morning’ and ‘definitely evening’). Compared with CC carriers, we also found that homozygous carriers of the G risk allele (i.e. GG) had a higher risk of T2D in all chronotype categories, except for ‘definitely evening’.

**Table 4:** (a) Odds ratios and 95% CIs for the association between *MTNR1B* rs10830963 genotype and type 2 diabetes, separated by chronotype. (b) Odds ratios and 95% CIs for the association between chronotype and type 2 diabetes, separated by the *MTNR1B* rs10830963 genotype

rs10830963 genotype	Chronotype			
	Definitely morning	More morning than evening	More evening than morning	Definitely evening
	OR (95% CI)	OR (95% CI)	OR (95% CI)	OR (95% CI)
(a)				
CC (reference group)	1	1	1	1
CG	1.17 (1.08, 1.25)	1.11 (1.04, 1.18)	1.05 (0.98, 1.12)	1.11 (0.97, 1.27)
GG	1.25 (1.10, 1.42)	1.24 (1.10, 1.39)	1.20 (1.06, 1.35)	1.10 (0.87, 1.39)

Chronotype	rs10830963 genotype		
	CC	CG	GG
	OR (95% CI)	OR (95% CI)	OR (95% CI)
(b)			
Definitely morning (reference group)	1	1	1
More morning than evening	0.98 (0.92, 1.05)	0.93 (0.86, 1.00)	0.99 (0.84, 1.17)
More evening than morning	1.11 (1.03, 1.19)	1.01 (0.93, 1.08)	1.10 (0.93, 1.29)
Definitely evening	1.27 (1.16, 1.40)	1.23 (1.10, 1.38)	1.16 (0.90, 1.49)

Logistic regression analysis adjusted for age, sex, self‐reported sleep duration, insomnia, BMI, systolic blood pressure, smoking, alcohol intake frequency, test centre, principal components of ancestry and Townsend index.

In the next step, we separately examined the association between chronotype and the risk of T2D for CC, CG and GG carriers (Table 4b). Amongst participants with the genotype CC (nonrisk allele carriers), the late chronotype categories (i.e. ‘more evening than morning’ and ‘definitely evening’) were associated with a higher risk of T2D, compared with the reference group ‘definitely morning’. Whereas ‘definitely evening’ also conferred a higher risk of T2D amongst CG carriers, no significant differences in the risk of T2D were noted between chronotype categories amongst GG carriers.

## Discussion

The present study based on data from the UK Biobank found that both late chronotype and carrying the *MTNR1B* G risk allele were associated with an increased risk of type 2 diabetes (T2D). No significant interaction between chronotype and the *MTNR1B* rs10830963 genotype was observed. Additional analyses within the groups of CC and CG carriers showed that individuals with an evening chronotype had a higher risk of T2D, compared with those who reported morning chronotype. Importantly, no such difference in the risk of having T2D was observed for GG carriers when contrasting early and late chronotype. Our results differ from findings based on the Multi‐Ethnic Study of Atherosclerosis (MESA). By utilizing data from 1,513 subjects, the study showed that the risk of having T2D was higher amongst *MTNR1B* risk allele carriers who were early risers, compared with carriers who were late risers 6. One explanation for these discrepant results could be that chronotype in the UK biobank was based on self‐reports. Self‐report assessments of chronotype can be subject to reporting bias. In MESA, chronotype was instead objectively measured by 7‐day actigraphy registration.

In addition to the association between the *MTNR1B* risk allele and T2D, we found that chronotype was associated with the risk of T2DM. This association was also significant when adjusting for the rs10830963 risk allele. Compared with those reporting a definitely morning chronotype, participants reporting a more evening than morning chronotype or definitely evening chronotype exhibited higher odds of T2D. Different mechanisms could explain the association between evening chronotype and T2D. These include the desynchrony between behavioural patterns (feeding, sleep) and endogenous circadian rhythms controlling glucose metabolism 16, 17, preferences for calorie‐dense food with higher sucrose and saturated fatty acids 18 and increased artificial light exposure during evening hours 19.

Overall, the pattern that tendency towards eveningness is associated with higher odds of having T2D is in line with results from a previous study utilizing the UK Biobank cohort. In this study, the researchers systematically investigated the association between chronotype and morbidity and mortality of various diseases 9. One main finding of the previous study was that those who were definite evening types were significantly more likely to have diabetes compared with those who were definite morning types. However, in our analysis, in addition to sex and age (which were used as confounders in the previous report), we also adjusted for additional confounders, such as sleep duration and blood pressure. Moreover, in the present study, probable T2D was defined using a validated algorithm based on self‐reported disease, medication and T2D diagnosis in medical history 13. In the previous report, data on diabetes were derived from self‐reports, including ‘diabetes’, ‘gestational diabetes’, ‘type 1 diabetes’ and ‘type 2 diabetes’ 9. Collectively, whereas the previous study showed an association between late chronotype and diabetes, here we further demonstrate a specific risk of late chronotype and *MTNR1B* genotype with T2D.

Altogether, our findings suggest that both carrying the *MTNR1B* risk allele and being a late chronotype may independently increase the risk of T2D. Assessing chronotype and the *MTNR1B* risk allele may be therefore useful in the risk estimation of T2D. Adherence to a lifestyle that is associated with earlier‐phased circadian rhythms, such as regular bright light exposure and physical activity during morning hours 20, 21, may help reduce the risk of developing T2D amongst CC and CG carriers. In contrast, GG carriers may not meaningfully reduce their risk to develop T2D by altering their circadian profile. Our study is limited by its cross‐sectional design. Thus, causality cannot be inferred from the associations observed. Finally, it is worth noting that the prevalence of T2D in the UK Biobank population (4.6%) is lower compared with the general population of the UK and other European countries 22, 23.

## Conflicts of Interest

The authors declare no conflict of interest. The funders had no role in the design of the study; in the collection, analyses or interpretation of data; and in the writing of the manuscript or in the decision to publish the results.

## Author Contributions

XT and CB conceived and designed the study. XT, DC and CB performed the statistical analyses. XT, HBS and CB interpreted the results. XT, DC., HBS and CB drafted the manuscript.

## Supporting information


**Table S1.** Odds ratios and 95% CIs for type 2 diabetes, separated by rs10830963 genotype and chronotype in complete case analysis (*n* = 297 091) (Nagelkerke, 1991).
