# Supplementary material for: Associations between chronotype, MTNR1B genotype and risk of type 2 diabetes in UK Biobank
Source: J Intern Med. 2019 Nov 6;287(2):189–96. doi: 10.1111/joim.12994 (PMC7003850; doi:10.1111/joim.12994)
Supplement: Supplementary file 1 — Table S1. Odds ratios and 95% CIs for type 2 diabetes, separated by rs10830963 genotype and chronotype in complete case analysis (n = 297 091) (Nagelkerke, 1991). [file JOIM-287-189-s001.docx]

**Table S1:** Odds ratios and 95% CIs for type 2 diabetes, separated by rs10830963 genotype and chronotype in complete case analysis (n=297,091) (Nagelkerke, 1991)

|  | **Model 1** | |  | **Model 2** | |  | **Model 3** | |  | **Model 4** | |  |
| --- | --- | --- | --- | --- | --- | --- | --- | --- | --- | --- | --- | --- |
| **Exposure** | **OR (95% CI)** | **P*** | **R^2^** | **OR (95% CI)** | **P*** | **R^2^** | **OR (95% CI)** | **P*** | **R^2^** | **OR (95% CI)** | **P*** | **R^2^** |
| **rs10830963 genotype** |  |  |  |  |  |  |  |  |  |  |  |  |
| CC | 1 |  | .058 | 1 |  | .060 | 1 |  | .064 | 1 |  | .183 |
| CG | 1.10 (1.06, 1.15) | 2.1218E-7 |  | 1.11 (1.06, 1.15) | 2.054E-7 |  | 1.10 (1.06, 1.15) | 2.7053E-7 |  | 1.10 (1.06, 1.15) | .000001 |  |
| GG | 1.19 (1.11, 1.27) | 5.2768E-7 |  | 1.19 (1.11, 1.27) | 4.3418E-7 |  | 1.19 (1.11, 1.27) | 5.1553E-7 |  | 1.21 (1.13, 1.29) | 1.3818E-7 |  |
|  |  | |  |  | |  |  | |  |  | |  |
| **Chronotype** |  |  |  |  |  |  |  |  |  |  |  |  |
| Definitely morning | 1 |  | .060 | 1 |  | .060 | 1 |  | .066 | 1 |  | .183 |
| More morning than evening | 0.85 (0.81, 0.89) | 1.0681E-12 |  | 0.85 (0.81, 0.89) | 1.2511E-12 |  | 0.84 (0.80, 0.88) | 1.8517E-13 |  | 0.96 (0.92, 1.01) | .108 |  |
| More evening than morning | 1.01 (0.96, 1.05) | .851 |  | 1.01 (0.96, 1.06) | .829 |  | 0.99 (0.95, 1.04) | .808 |  | 1.07 (1.02, 1.12) | .011 |  |
| Definitely evening | 1.37 (1.28, 1.46) | 1.7652E-21 |  | 1.37 (1.28, 1.46) | 1.3064E-21 |  | 1.34 (1.25, 1.43) | 2.113E-18 |  | 1.26 (1.17, 1.35) | 5.2121E-11 |  |
|  |  |  |  |  |  |  |  |  |  |  |  |  |

Chronotype × rs10830963 genotype interaction regarding risk of type 2 diabetes OR (95 CI%): 0.97 (0.94, 1.00), P=.085 (Model 4); 0.98 (0.95, 1.01), P=.195 (adjusting for age, sex, chronotype, and rs10830963 genotype).

*Compared to the reference group (derived from logistic regression analysis). In order to compare the goodness-of-fit between statistical models, we computed the Nagelkerke’s R-squared value for each logistic regression model. A higher R-squared value reflects better fitness of the logistic regression model (Nagelkerke, 1991).

*Model 1*: adjusted for age and sex.

*Model 2*: adjusted for confounders in Model 1 + self-reported chronotype (or rs10830963 genotype when investigating the association between self-reported chronotype and T2D).

*Model 3*: adjusted for confounders in Model 1 + self-reported sleep duration + insomnia.

*Model 4*: adjusted for confounders in Model 2 + self-reported sleep duration + insomnia + BMI + systolic blood pressure + smoking + alcohol intake frequency + test center + principal components of ancestry + Townsend index.
